# Finding an Optimal Corneal Xenograft Using Comparative Analysis of Corneal Matrix Proteins Across Species

**DOI:** 10.1038/s41598-018-38342-4

**Published:** 2019-02-12

**Authors:** R. Sharifi, Y. Yang, Y. Adibnia, C. H. Dohlman, J. Chodosh, M. Gonzalez-Andrades

**Affiliations:** 1 Massachusetts Eye and Ear and Schepens Eye Research Institute, Department of Ophthalmology, Harvard Medical School, Boston, USA; 2 Department of Ophthalmology and Visual Sciences, University of Toronto, Toronto, Canada; 3 School of Medicine, Yeditepe University, Istanbul, Turkey; 4 Maimonides Biomedical Research Institute of Cordoba (IMIBIC), Department of Ophthalmology, Reina Sofia University Hospital and University of Cordoba, Cordoba, Spain

## Abstract

Numerous animal species have been proposed as sources of corneal tissue for obtaining decellularized xenografts. The selection of an appropriate animal model must take into consideration the differences in the composition and structure of corneal proteins between humans and other animal species in order to minimize immune response and improve outcome of the xenotransplant. Here, we compared the amino-acid sequences of 16 proteins present in the corneal stromal matrix of 14 different animal species using Basic Local Alignment Search Tool, and calculated a similarity score compared to the respective human sequence. Primary amino acid structures, isoelectric point and grand average of hydropathy (GRAVY) values of the 7 most abundant proteins (*i.e*. collagen α-1 (I), α-1 (VI), α-2 (I) and α-3 (VI), as well as decorin, lumican, and keratocan) were also extracted and compared to those of human. The pig had the highest similarity score (91.8%). All species showed a lower proline content compared to human. Isoelectric point of pig (7.1) was the closest to the human. Most species have higher GRAVY values compared to human except horse. Our results suggest that porcine cornea has a higher relative suitability for corneal transplantation into humans compared to other studied species.

## Introduction

Corneal transplantation is one of the most successful organ transplantations with over 180,000 surgeries performed annually^1^. However, the need for donor corneas far exceeds the current corneal supply, especially in resource-poor countries. Over 10 million worldwide untreated patients are estimated to be waiting for corneal transplant^2^. This has fueled interest in the scientific community to search for an alternative solution to corneal allograft surgery, ranging from tissue engineering^3,4^ and regenerative medicine^5,6^ to decellularized corneal xenografts^7,8^. Although notable progress has been made to develop synthetic bioengineered scaffolds^5,6^, they are so far incapable of mimicking the biomechanical properties and molecular microarchitecture of the native tissue^9^. In addition to enhancing properties of the bioengineered scaffold, some prior studies have concurrently focused on application of xenogeneic corneal tissue in humans^10,11^.

The advantages of using xenogeneic tissues over synthetic scaffold are: (i) the close similarity of chemical composition and microarchitecture of xenogeneic tissue with human cornea, (ii) accessibility, (iii) lower cost, and (iv) their analogous optical and biomechanical properties to those of the human cornea^12^. However, despite anatomical, biomechanical and chemical similarities of xenograft with human cornea, the main challenge associated with their application has been antigenicity^10,13,14^. Resident cells within the extracellular matrix (ECM) of the xenogeneic tissues can trigger innate and adaptive immune responses, inducing xenograft rejection. One of the main reasons for such immune response is the presence of different antigens, such as Galα1,3Ga, for which humans have natural antibodies that lead to acute graft rejection^15^. However, even in gal-epitope knock out models, a humoral response against xenogeneic tissues is still observed, suggesting the involvement of other antigens in the immune-mediated response^16^.

To overcome this immunological barrier, decellularization of xenogeneic cornea has been recently proposed as a strategy to remove cellular antigens from the tissue while preserving the biological scaffold^17^. Various techniques have been developed for this purpose including chemical, physical, and enzymatic treatments^11^, which seek to maintain a balance between preserving matrix compositions and removing all cells and cellular debris from the xenograft^18–20^. This allows for preservation of the biomechanical and optical properties of the xenogeneic cornea, while minimizing the inflammatory response associated with antigenic nature of xenogeneic components^13^. However, the decellularization process does not eliminate 100% of antigenic components, and the remaining constituents have been shown to still elicit an immune response^21^. Even the remaining extracellular matrix that will serve as scaffold for corneal replacement, may differ from the host in terms of protein composition and structure, which can act as antigens that stimulate an immune response^22^. It is, therefore, important to select the best animal model in order to minimize immune response and improve outcome of the xenotransplant. In this regard, corneas from non-human primates such as gibbon, which are most genetically similar to human, have been used as donor grafts in humans^23^. Although the results were promising, the high risk of infection, cost of raising herds in large numbers, and behavioral similarities to humans makes the practice questionable and, thus, renders them unlikely candidates for this application^24^.

Although numerous animals, including pigs, sheep, dogs, rabbits, cows and fish, have been used as sources of corneal tissue for decellularization, the selection of an appropriate animal model must take into consideration not only the anatomical characteristics, availability and economical feasibility, but also the similarity of protein structures to those of human. Although protein composition of human cornea has been studied^25^, there is little known about the differences in the composition and structure of corneal proteins between humans and other animal species. We aim to investigate the differences in corneal matrix protein composition and their characteristics between various species and humans to determine the most appropriate animal model for corneal decellularization process.

## Results and Discussion

Corneal integrity and clarity are requisites for vision. Given the prevalence of corneal pathologies (e.g. chemical trauma, infections and corneal dystrophies) and the shortage of donor corneas, there is an urgent need to develop alternatives such as tissue engineering-based and decellularized corneas. The key for success in xenogeneic corneal transplant is not only preservation of mechanical and optical properties of the intact cornea, but also reduction in antigenicity of corneal components that lead to host immune responses^15^. Although decellularization techniques have been shown to remove cells and their debris from xenogeneic tissue, the remaining protein scaffolds also express antigens, susceptible to host immune cell recognition^26^. Antigenicity has been found to be closely linked with the primary and secondary structures of the protein, which depends on the amino acid sequence. Thus, antigen-antibody recognition and subsequent specificity of immune response can be predicted without having direct evidence for the protein tertiary structure^22^. The analysis of amino acid sequence of proteins constituting the corneal stroma and its comparison between different species can lay a foundation for the selection of the right xenogeneic tissue to minimize antigenicity and enhance the outcome of the transplant.

Xenotransplantation has been reported as early as 17^th^ century, when blood transfusions were carried out between animals and humans^27^. Earlier attempts of organ transplants including heart and kidney have focused on primates, which are more immunologically similar^28^. Non-primate animals such as rabbit, pig and goat have been used on occasion but with little success, with rejection often occurring in hours to days^28^. With the advances in technology and research, non-primate organs have been successfully transplanted into humans. For instance, the first pig valves that were implanted into humans were limited by the host immune response, and only 45% of the valves were functioning at 1 year^29^. By eliminating soluble proteins, denaturing glycoproteins and fixing the valves in glutaraldehyde solution, the success of functioning valve significantly increased, becoming a widely available option for human heart valve replacement^29^. Regarding the cornea, there have been several attempts to implant corneal xenografts in humans. The first case reported by Kissam in 1838 used a cornea from a 6-month-old pig, which failed within 2 weeks^30^. Other reports involving sheep, dog, rabbit and fish had poor results with most grafts rejected within a month^23^. The most successful graft involved gibbons, a closely related specie to humans, where 50% of corneas remained transparent for >5 months.

Despite genetic variations, corneal differences between species can be determined according to structural^31^ and chemical properties^32^. Structural comparisons of corneal tissues have been done using second harmonic generation microscopy^31,33–35^. It was shown that the anterior stroma of different species such as pig, cow, rabbit, rat, chicken and human has similar interwoven short bands of collagen. Compare to the anterior stroma, however, the central and posterior stromal lamellae have longer collagen bundles with a denser packing, which are primarily parallel to the corneal surface and have distinct spatial distributions for different species^31^. For instance, the collagen bundles in bovine and porcine corneas were interwoven, while the orientation of fibers in chick cornea has a gradient that changes with depth. In contrast, the central and posterior parts of the human corneal stromal lamellae have an orthogonal arrangement of collagen fibrils, aligned between the superior-inferior and nasal-temporal meridians, with an elevated number of fibers towards the periphery^36,37^. However, the collagen lamellae in the anterior stroma of human have a random alignment with an even distribution^33^. Such organizations can impact the structural properties of the cornea and are important factors in determining the suitability of the explant in terms of mechanical and optical properties, but not antigenicity and stability, which are dictated by the chemical composition of the transplant. On the other hand, Watanebe *et al*. used lysis and electrophoresis of the corneal tissues to show that the soluble corneal proteins differ among the various animal species and play an important role in the antigenicity of tissue^38^. Until now, several approaches including radioimmunoassay chemistry^32^, BEOracle (B-Cell Epitope Oracle)^22^, BCPred (B-cell epitope prediction server)^39^, BepiPred (sequential B-Cell epitope predictor)^40^, and Hopp-Woods method^41^, have been used to predict the antigenicity of proteins. Most of the recent approaches relied on the amino-acid sequence of the proteins.

### Basic Local Alignment Search Tool (BLAST)

Taking advantage of the protein primary structures, we applied BLAST^42^ to identify and compare the amino-acid sequences of 16 proteins present in the corneal stromal matrix of 14 different animal species, and calculated a similarity score compared to the respective human sequence (Table 1). The BLAST algorithm works based on a heuristic method through locating short matches between the two sequences (seeding), finding local alignments, and then approximating the similarity between the two sequences^43^. In terms of collagens (i.e. α-1(I) chain, α-2(I) chain, α-1(III) chain, α-1(V) chain, α-2(V) chain, α-1(VI) chain, α-2(VI) chain, α-3(VI) chain and α-1(XII) chain), which constitute almost 79% of the corneal stromal extracellular matrix, among the proteins studied for this report, pig (93.8%) was the most similar species to human, with the cat (92.7%) and sheep (91.5%) in second and third places respectively. However, in term of non-collagenous proteins (i.e. decorin, lumican, keratocan, biglycan, MAM domain-containing protein, prolargin and vimentin), which comprise 21% of the corneal stromal extracellular matrix, horse with 92.5% was the most similar to human, with the dog (90.5%) and cat (90.3%) in the second and third places, respectively. The amino acid sequence of zebrafish, on the other hand, was the least similar to human in terms of both collagenous and non-collagenous proteins. Most of the other species had the similarity distribution between 70% to 90%.

**Table 1:** Similarity of each protein’s primary sequence compared to human (%), including the Total Protein Sequence Similarity Score (TPSSS) and relative abundance of protein in human corneas (according to Dyrlund *et al*.)^25^.

	Collagen alpha-1(I) chain (COL1A1)	Collagen alpha-2(I) chain (COL1A2)	Collagen alpha-1(III) chain (COL1A3)	Collagen alpha-1(V) chain (COL1A5)	Collagen alpha-2(V) chain (COL5A2)	Collagen alpha-1(VI) chain (COL6A1)	Collagen alpha-2(VI) chain (COL6A2)	Collagen alpha-3(VI) chain (COL6A3)	Collagen alpha-1(XII) chain (COL12A1)	Decorin	Lumican	Keratocan	Biglycan	MAM domain-containing protein 2 (MCMDC2)	Prolargin	Vimentin	TPSSS (%)
Pig	97	94^a^	92^a^	95^a^	92^a^	92^c^	72^b^	88^c^	94*	86.5	89^a^	93^a^	88*	90	94^b^	98	91.80
Cat	93.5^d^	94^b^	93	95	92	91^b^	90.6^d^	85^b^	95^d^	89^b^	90^b^	92^a^	89	92^d^	91	98	91.18
Sheep	93^b^	92^b^	91^b^	93^b^	92^b^	90^b^	86^d^	85^c^	94^d^	86	89^b^	92^b^	88^a^	89^d^	93^b^	98^c^	89.95
Dog	97^a^	94^a^	93^b^	82^b^	92^b^	32^a^	90.6*	82^b^	96^d^	89	91^b^	92^a^	88	92^d^	94^b^	98	89.87
Goat	93^b^	92^b^	91^b^	95^b^	92^b^	91^b^	92^d^	80.6^d^	94^d^	86	89^b^	92^a^	88^b^	89^b^	93^b^	98^b^	89.85
Cow	89	92^a^	89	99^c^	89.5	91^a^	92^a^	84	94^a^	87	88^a^	92	88^a^	89^b^	93^a^	98	88.87
Rabbit	92*	93^a^	92^b^	94^b^	91^b^	90^b^	94^b^	62^b^	93	88	91^a^	90^a^	88^a^	92.5^d^	94^b^	97^b^	88.56
Mouse	92	90^a^	67^c^	94^a^	90^a^	90^a^	91^a^	84	94^a^	78	87^a^	86	89	90^a^	89^a^	97	87.87
Guinea pig	91.5^d^	92^a^	42^d^	94^b^	90^b^	83	91.5^d^	77.8^d^	91.5^d^	88	83^b^	84^a^	89^a^	90^b^	91^b^	96	87.79
Rat	92	91^a^	90^a^	94^a^	90^a^	89^b^	85	71.2^d^	94^c^	75	85^a^	86^a^	89	88	89^a^	97^b^	86.83
Horse	92^b^	65^a^	96^c^	99^b^	93^b^	89^b^	91^b^	83^b^	88^c^	89	99^a^	92^d^	88	92^b^	92^b^	92^b^	83.08
Chick	97^a^	83^a^	73^a^	88^a^	87^d^	69^a^	71^a^	62^a^	81	77	67^a^	70	51	76	79	88	81.82
Zebrafish	78	71^a^	73*	92^c^	70^a^	55^b^	52^c^	46^d^	63^c^	63	93^a^	62^a^	63	55^a^	55^a^	55^a^	69.68
Relative abundance of protein in human corneas^25^ (%)	20	17.2	0.5	1.1	1.3	2.2	1.7	4.7	1.6	5.1	3.5	3.4	0.6	0.3	0.2	0.2	—

*(Asterisk) indicate protein sequences that were not available in the database. This was estimated according to species that were most genetically similar.

^a^Precursor

^b^Predicted

^c^Partial

^d^Isoforms.

**Figure 1:**
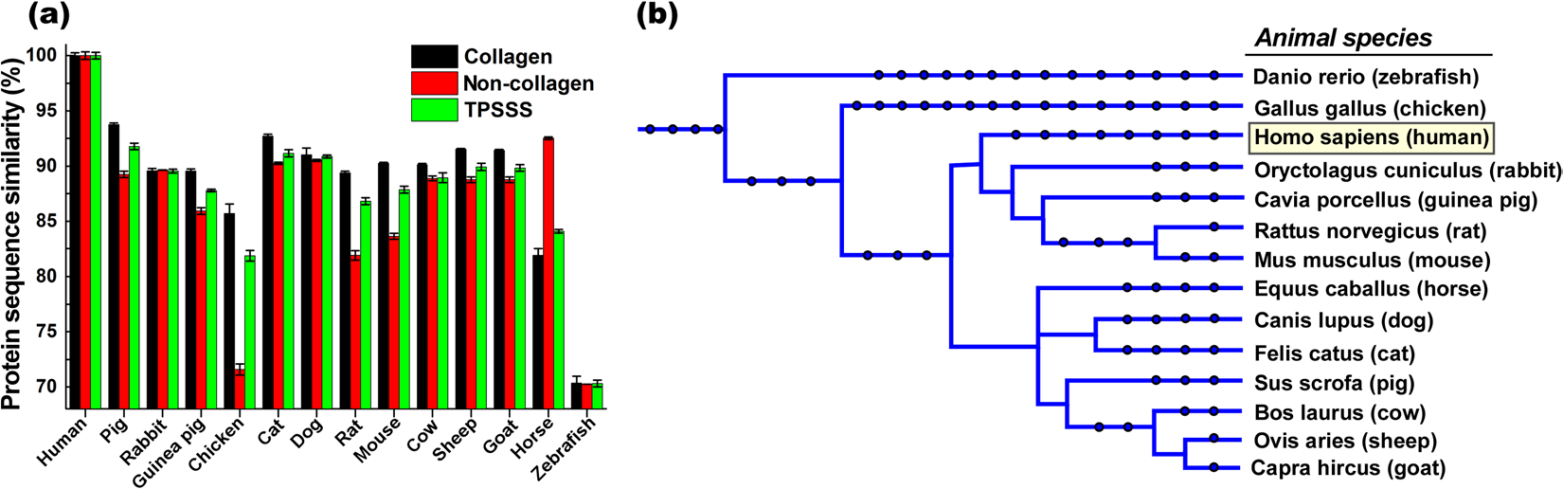
Collagen, non-collagen, and total protein sequence similarity score (TPSSS) of extracellular matrix in the corneal stroma of different species with respect to those of human (**a**). Phylogenetic tree of studied species (**b**).

In the total calculated score, taking into account all corneal proteins examined and their abundance, the pig had the highest score (91.8%) suggesting most similarity of corneal stromal matrix to human, while zebrafish had the lowest scores (69.7%). While the cat had the second highest similarity to the human with a total score of 91.2%, the similarity scores of other species lay in a narrow range of 81.8% to 89.9% (Table 1 and Fig. 1a). The phylogenetic comparison^44,45^ of these species also exhibited a similar trend, suggesting that the species most closely related to human have higher similarity scores (Fig. 1b). For instance, pig and cat with the highest scores were closer to human comparatively, while zebrafish and chicken with the lowest scores were the most distant. Rabbit, guinea pig, rat and mouse are more closely related to human than horse, dog, cat, pig, sheep, goat and cow, and higher sequence scores of those species was expected. However, convergent evolution^46^ might act on or against such relationships expressed by the phylogenetic tree, and contribute to such protein composition similarities. As the species become more distant to human such as chicken and zebrafish, convergent evolution becomes less pronounced and the similarity of primary sequence in the constituent proteins is reduced, following the phylogenetic relationships (Fig. 1b).

### Protein Primary Structure Analysis

**Figure 2:**
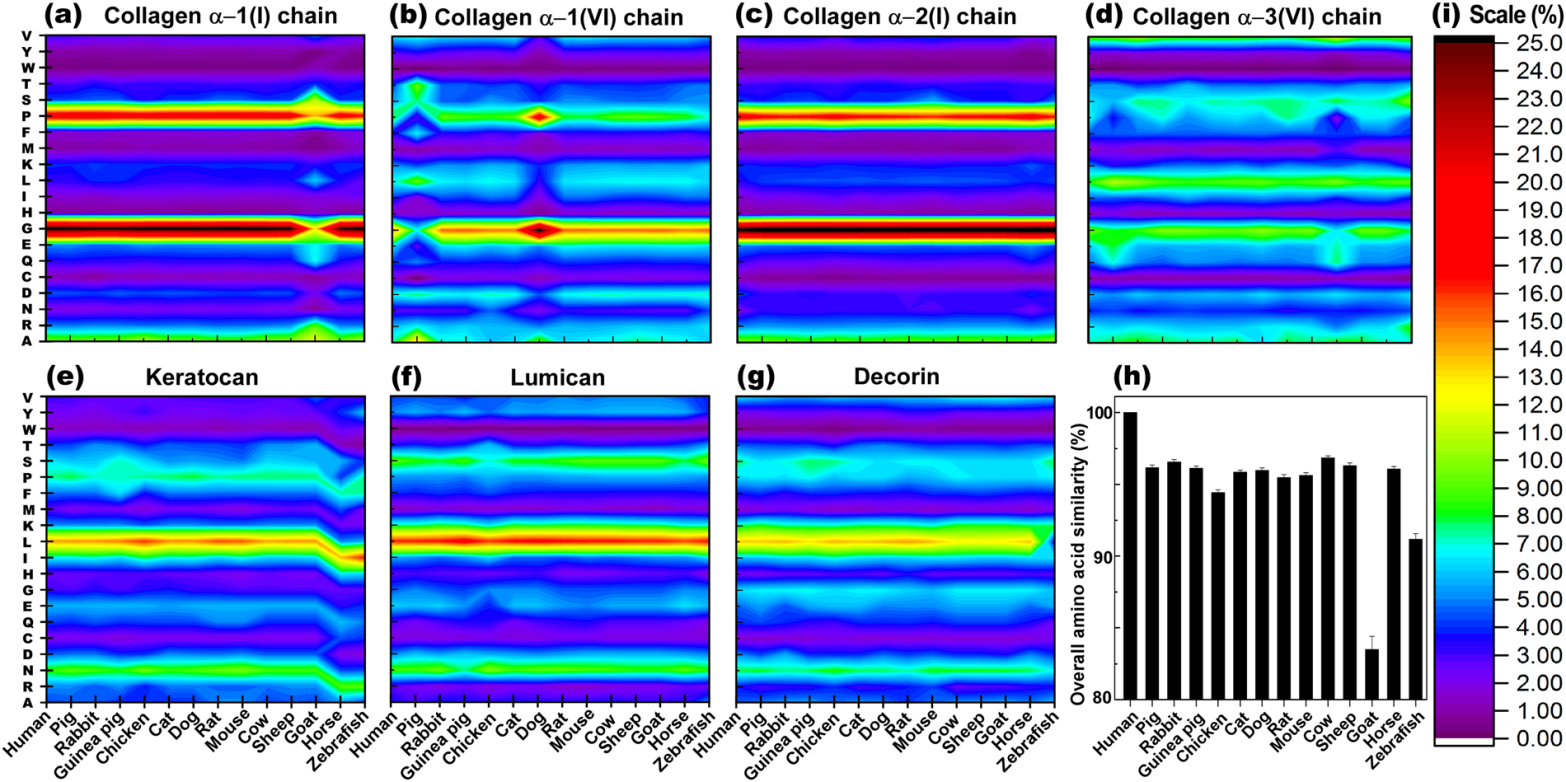
Protein primary structure comparison of seven most abundant proteins in the extracellular matrix of corneal stroma between different species. (**a**–**g**) The overall amino acids similarity of proteins in the extracellular matrix of corneal stroma among different species (**h**) (Y axes show the amino acids list, and color scale (**i**) illustrates the abundance of amino acid).

Despite the fact that BLAST locates the homologous sequences through seeking and comparing a sequence of interest between two proteins, thusly providing valuable information about the similarity of large biomolecules, it leaves gaps when there are poor alignments in the sequence^42^. Moreover, while some changes in amino acid sequence might not have an effect in protein functions (i.e. hydrophobic with another hydrophobic, cationic with another cationic, etc.), other changes might significantly alter the protein structure and function (i.e. hydrophobic with hydrophilic or cationic with anionic, etc.). BLAST cannot distinguish such changes in the analysis. We envisioned that the sequence comparison of proteins according to total amino acid constituent abundance may fill such gaps and offer another view to the chemical composition of the analyzed proteins. Such analysis takes into account the abundance of each amino acid in the structure of each protein and/or the entire cornea, and can be used as a complementary tool to compare the similarities of those proteins among different species. Accordingly, the primary amino acid structures of the 7 most abundant proteins in the corneal stroma (*i.e*. collagen α-1 (I), α-1 (VI), α-2 (I) and α-3 (VI), as well as decorin, lumican, and keratocan) were extracted and compared to those of human (Fig. 2).

Our analysis revealed that in terms of collagen α-1 (I), while the abundance of amino acids was relatively similar among the species, goat had significantly lower glycine and proline content (Fig. 2a), suggesting a reduced tendency of the protein strand to form a tight turn. Moreover, goat expressed higher serine and arginine content compared to those of human, leading to an enhanced hydrogen bonding (H-bonding) capability and higher hydrophilicity. These changes drastically influence not only primary, but also higher hierarchy of protein structures along with their function and consequently, may reduce the suitability of those tissues for transplantation in the human. Primary amino acid analysis also revealed that collagen α-1 (VI) in pig had lower glycine, proline, and isoleucine, and higher alanine and threonine content compared to those of human and many other species (Fig. 2b). These changes can also hinder the formation of tight turns in the protein, while affecting hydropathicity and H-bonding capability. Conversely, collagen α-1 (VI) of dog exhibited higher glycine, proline, and alanine, and lower isoleucine percentage, promoting a tight-turn conformation of the protein, without altering hydropathicity, as the elevated alanine can be compensated with depressed isoleucine contents. However, for collagen α-2 (I) and lumican (Fig. 2c,f), the amino acid analysis did not show significant variations between species, indicating that the structures of these proteins are more conserved. Despite similar content of collagen α-3 (VI) between species, pig and goat had slightly lower proline and higher glutamine content, restraining tight-turn formation, while promoting H-bonding with an increased hydrophilicity (Fig. 2d). Moreover, collagen α-3 (VI) in zebrafish had greater serine and lysine percentages, indicating elevated H-bonding capacity and enhanced hydrophilicity, while carrying more positive charge. In the case of keratocan, although most species, including human, have relatively similar amino acids compositions, zebrafish and horse significantly differed from the rest of the species (Fig. 2e).

Zebrafish and horse expressed higher arginine, histidine, cysteine, isoleucine, phenylalanine, and lower asparagine, aspartic acid, leucine, lysine and threonine. Although reduced lysine (positively charged) can be partially compensated with excess of arginine (positively charged), higher histidine and reduced aspartic acid alter the overall charge of the protein, making it more positive. Similarly, the lack of leucine and phenylalanine (both hydrophobic) can be balanced with extra isoleucine and threonine; less asparagine shifts the hydropathicity of the protein towards hydrophobic. Keratocan in zebrafish also exhibited greater tryptophan, which further enhances hydrophobicity. Additionally, deficiency of cysteine in both zebrafish and horse influences the capacity of the protein for disulfide bonding, and adversely affects three-dimensional structure of keratocan and its stability, making it more susceptible to degradation. On the other hand, decorin in zebrafish is more hydrophilic due to the lower content of leucine (Fig. 2g). Taking into account the abundance of protein in the cornea of different species and their amino acid constituents, it appeared that most species have relatively similar amino acid composition with the exception of zebrafish, goat, and chicken (Fig. 2h). While the higher content of isoleucine in zebrafish can be balanced with lower leucine, the substantial deficiency of proline significantly affects the tight-turn formation and high hierarchy structure of protein. Goat on the other hand, in general lacks sufficient amount of not only proline but also glycine, where both are important for tight-turn formation. These analyses are in good agreement with BLAST primary sequencing and consistent with the phylogenetic tree, indicating that the species most close to human have higher similarities. However, it is interesting that all species we studied had a lower proline content compared to human, which restricts the protein strand from forming tight-turns and impacts protein folding and consequent secondary and tertiary protein structures.

### Isoelectric point (PI) analysis

**Figure 3:**
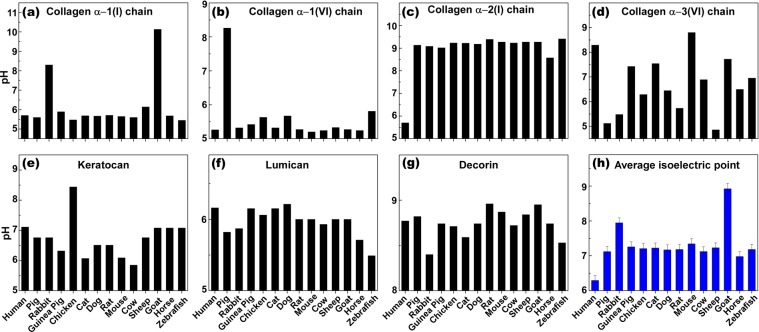
Isoelectric point comparison of the most abundant proteins in the extracellular matrix of corneal stroma among different species. (**a**–**g**) The average isoelectric point of proteins in the extracellular matrix of corneal stroma among different species (**h**).

The isoelectric point (PI) of protein is the pH at which a macromolecule does not carry an electrical charge, and is dictated by the prevalence of amino acids with positively or negatively-charged side chains in the protein structure. Although PI greatly affects the intramolecular interactions in secondary and tertiary structures (and quaternary when relevant), PI also impacts the intermolecular interactions of protein with protein (e.g., antigen-antibody interactions), DNA (e.g., gene expression) and any other molecular interactions. Since the PI can drastically affect H-bonding, hydrophilic and electrostatic interactions, its variation between species may affect the cell-scaffold interactions and consequently biocompatibility of the scaffold. Additionally, it has been shown that the PI is linked with the antigenicity of the protein^47^. Therefore, comparisons of the PI for the proteins (7 most abundant) constituting corneal stroma of different species complements the primary sequence (BLAST) approach to find the most similar xenogeneic tissue for corneal transplantation in humans. Figure 3a–g shows the PI of individual proteins for all species we studied. Collagen α-1 (I) chain in rabbit and goat, owing to higher content of basic amino acids, has a higher PI value with respect to the other species, making the protein positively-charged at physiological pH (Fig. 3a). For collagen α-1 (VI), which constitutes 1.1% of corneal stromal protein, pig has a relatively higher PI value compared to the other species (Fig. 3b). Collagen α-2 (I) in all species has higher PI values compared to human, due to greater content of basic amino acids such as arginine (Fig. 3c). In case of collagen α-3 (VI), almost an opposite pattern was observed, except for mouse, which stemmed from lower content of arginine and lysine (Fig. 3d). The PI of keratocan in chicken is comparatively higher than that of the other species, also due to different ratios of basic and acidic amino acid content (Fig. 3e). All species expressed a similar PI for the lumican with an exception of zebrafish that showed lower PI value and less basicity (Fig. 3f). Taking into account the abundance of each protein and the PI of individual proteins for the species of interest, we noticed that the PI values of species fell within a relatively narrow range of 7–7.5 with an exception of rabbit (7.9) and goat (8.9) which had much higher PIs, compared to that of human and other species. Moreover, the PIs of pig (7.1) and horse (7.0) were the closest to the human, suggesting overall similarity in net charge between stromal proteins of these species with human.

### Grand Average of Hydropathy (GRAVY) Value Analysis

**Figure 4:**
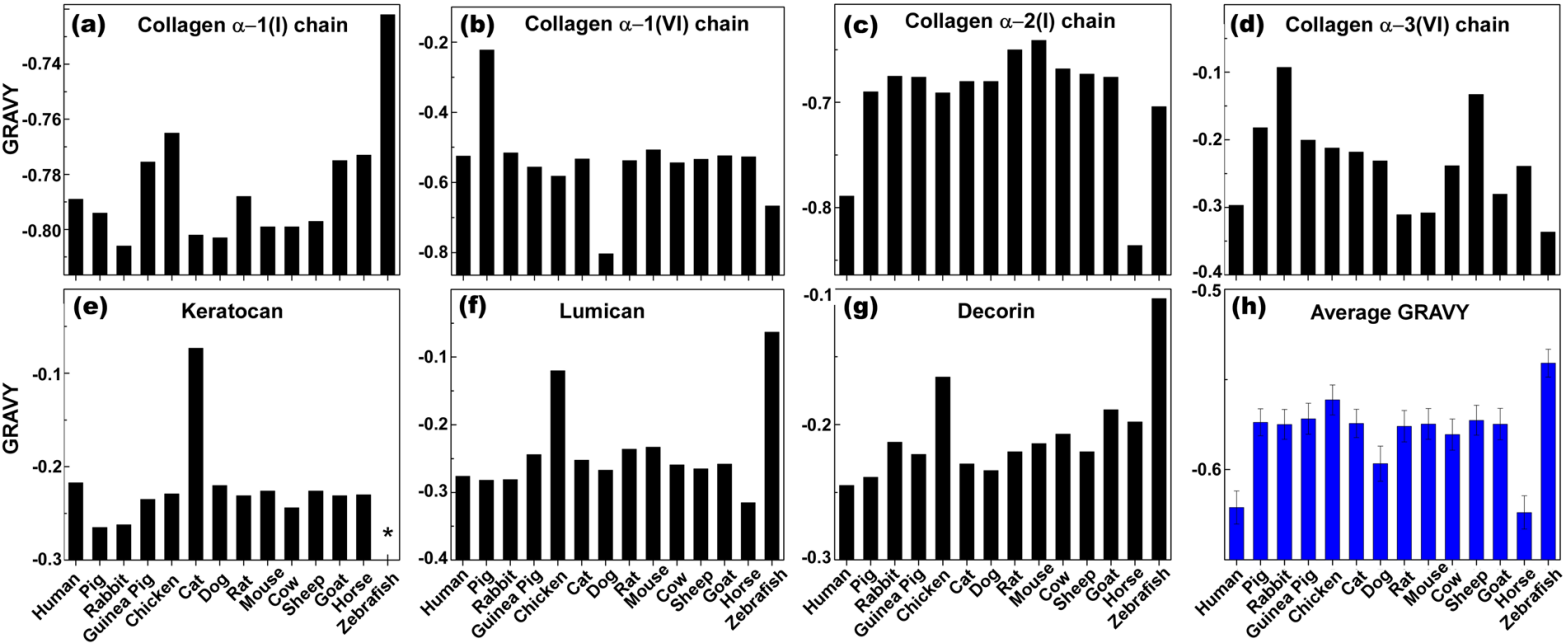
The grand average of hydropathicity (GRAVY) values comparison of the most abundant proteins in the extracellular matrix of corneal stroma among different species. (**a**–**g**) The average GRAVY values of proteins in the extracellular matrix of corneal stroma among different species (**h**). (*The data point was not available).

GRAVY values are calculated by summation of the hydropathy values of each amino acid residue, and divided by the length of the protein sequence, where the negative and positive values are indexed for hydrophilic and hydrophobic residues, respectively. Such analysis provides a larger picture about the hydrophilic/hydrophobic nature of the proteins, which plays a critical role in molecular level understanding of inter- and intramolecular interactions. GRAVY values enable the prediction of molecular level interactions of biological entities with targets of interest, such as antibody-antigen and cell-scaffold interactions. In addition, it has been shown that the hydrophobicity of a protein is intimately linked with its antigenicity, and the highest local average hydrophilicity point is situated within or adjacent to an antigenic determinant^48^. Figure 4 demonstrates the GRAVY values of individual proteins for all species we studied. Collagen α-1 (I) chain along with collagen α-2 (I) are the most hydrophilic proteins present in the stroma (Fig. 4a,c). Collagen α-1 (I) chain in most of species was slightly more hydrophilic compared to human, except in horse, goat, guinea pig, and chicken. Zebrafish showed the highest hydrophobicity (Fig. 4a). However, collagen α-2 (I) in most species was more hydrophobic compared to human with an exception of horse (Fig. 4c). For collagen α-1 (VI), most animals had similar GRAVY values with the exceptions of pig, which was more hydrophobic, and dog, which was more hydrophilic (Fig. 4b). Collagen α-3 (VI) chain in most species was more hydrophilic with an exception of mouse, rat and horse, which was close to that of human, along with zebrafish that was more hydrophilic (Fig. 4d). Keratocan in most species had similar hydropathicity, except in cat, which was more hydrophilic. (Fig. 4e). Lumican and decorin’s GRAVY values in most species were quite similar with the exception of those in chicken and zebrafish that had higher hydrophobicity (Fig. 4f,g). Although antigenicity is a local property of amino acid sequence, and use of integration GRAVY values alone might not offer a practical approach to predict the antigenicity of a protein, such integration data can offer valuable information regarding the general hydrophobicity of corneal stroma, and help to find the right xenogeneic tissue for human corneal transplantation. The overall analysis (Fig. 4h) demonstrated most species have higher GRAVY values compared to human with the exception of horse. Moreover, zebrafish and chicken had the highest hydrophobicity among the studied species, suggesting their unsuitability for xenogeneic corneal transplantation. The variations of amino acid content of proteins across species not only impacts the specific protein structure and its function, but also influences its intermolecular and intramolecular interactions in three-dimensional microenvironments, including xenografts and tissue engineered corneal scaffolds. As the cellular response to the microenvironment strongly depends on spatial interactions (hydrophilic/hydrophobic) between cell binding proteins and the hydrophilic/hydrophobic functional groups present in the xenogeneic scaffold, finding the right scaffold with similar hydropathicity might minimize unfavorable responses associated with transplantation of xenogeneic tissue, and its consequent rejection.

Once the xenograft is implanted into the human host, human cells might migrate and repopulate the corneal xenograft. In this context, a remodeling of the animal corneal stroma will occur, where human stromal proteins produced by human cells would interact with xenogeneic proteins. Those differences, as already shown in composition and structure between proteins, could determine functional changes after xenotransplantation that might affect vital corneal properties, such as transparency. The uniformity of diameter of collagen fibers and interfibrillar distance are important factors for transparency. Three helical alpha chains coil together to form a helical domain, which self-assemble in a staggered manner to form microfibrils^37^. Hydrophobic and electrostatic forces play an important role in collagen fibril formation by driving the molecules to assemble side by side^37^. Pig, with its higher hydrophobicity of collagen α-1 (VI), may show altered cell adhesion and function after xenograft implantation. Thus, more investigation is required to test viability of human corneal cells in xenogeneic grafts. Additionally, proteoglycans (i.e. lumican, keratocan and decorin) also play a role in the maintenance of interfibrillar distance and transparency^49^. The role for lumican in regulation of collagen fibril assembly has been shown in lumican knock out mice, which demonstrate disarrayed spatial arrangement of corneal collagen and larger fibril diameters^50^. Lumican was found to be more hydrophobic in chicken and zebrafish, which may affect the spatial arrangement of collagen matrix in the xenogenic graft. In macular corneal dystrophy, lack of keratocan results in decreased interfibrillar spacing, leading to corneal thinning and opacification^51^. We found that zebrafish and horse keratocan had lower levels of cysteine, which might make it more susceptible to degradation. This could affect transparency of the transplant if host fibroblasts are not able to replace keratocan at the same pace it is degraded.

The selection of an appropriate animal model must take into consideration the differences in the composition and structure of corneal proteins between humans and other animal species in order to minimize immune response and improve outcome of the xenotransplant. Despite of the role of amino acid composition, isoelectric point, and hydropathicity of proteins in antigenicity^38,47,48^, there are specific xenogeneic antigens that can initiate the immune response in humans, such as N-glycolylneuraminic acid (NeuGc) and Galα1,3Ga (α-gal epitope). The expression of these antigens is cell type-specific and dependent on the microenvironment. Porcine corneal epithelial, stromal and endothelial cells express NeuGc^52^. However, α-gal is only expressed by porcine corneal stromal cells, and corneal endothelial cells during inflammation^53,54^. Humans have natural antibodies that bind to these antigens, inducing an acute graft rejection^15,16,55^. Thus, regardless of the decrease in antigenicity based on the similarities of corneal matrix proteins between pigs and humans, and the removal of the porcine cells after decellularization, it is still necessary to objectively demonstrate the lack of expression of these antigens (i.e. by Western Blot, Mass Spectrometry, etc.) prior to clinical translation, in order to facilitate the survival of corneal xenografts in humans.

Overall, the present study compares the corneal extracellular matrix proteins among different species using BLAST, amino acid composition analysis, isoelectric point, and hydropathicity approaches. The integration of our results, along with anatomical similarity of some animal corneas to humans in terms of size and thickness (Table 2), in conjunction with issues of availability and economical feasibility suggest that decellularized porcine cornea may have higher relative suitability for corneal transplantation into humans compared to other studied species. This is based on the assumption that there is a critical structure-function relationship between the different ECM proteins and that these measured differences will have a clinical effect. The in silico nature of our study has limitations, including the limited availability of amino acid sequences for some proteins and for others, availability of only hypothetical forms, isomers, or partial sequences. Regardless, these data lay a foundation for understanding the importance of extracellular matrix proteins in xenotransplantation.

**Table 2:** Corneal measurements across species and the total protein sequence similarity score (TPSSS). NA: not available.

	Corneal horizontal diameter (mm)	Corneal vertical diameter (mm)	Central corneal thickness (μm)	TPSSS (%)
Human	11.7	10.6	536	100
Pig^3^	14.9	12.4	666	91.80
Cat^25^	16.5	16.2	755	91.18
Sheep^30,31^	22.4–27	15.4–19	619	89.95
Dog^26^	13–17	12–16	562	89.87
Goat^30,31^	22.4–27	15.4–19	741	89.85
Cow^29^	23.9	29.8	1015	88.87
Rabbit^21,22^	13.4	13	407	88.56
Mouse^28^	2.3–2.6	2.3–2.6	122–137	87.87
Guinea pig^23^	NA	NA	227	87.79
Rat^27^	5.8	5.8	159	86.83
Horse^32^	25.7–34	19.5–26.5	828	83.08
Chick^24^	9	9	400	81.82
Zebrafish^33^	<2	<2	20	69.68

## Methods

As decellularization removes most cellular components (including epithelium and endothelium), only corneal extracellular matrix proteins with an abundance of higher than 0.2% w/w of corneal stroma were included in our analysis^25^. The selected proteins were collagens (*i.e*. α-1(I) chain, α-2(I) chain, α-1(III) chain, α-1(V) chain, α-2(V) chain, α-1(VI) chain, α-2(VI) chain, α-3(VI) chain, and α-1(XII) chain), proteoglycans (*i.e*. decorin, lumican, keratocan, and biglycan) and extracellular matrix proteins (*i.e*. MAM domain containing protein 2, prolargin, and vimentin).

**Figure 5:**
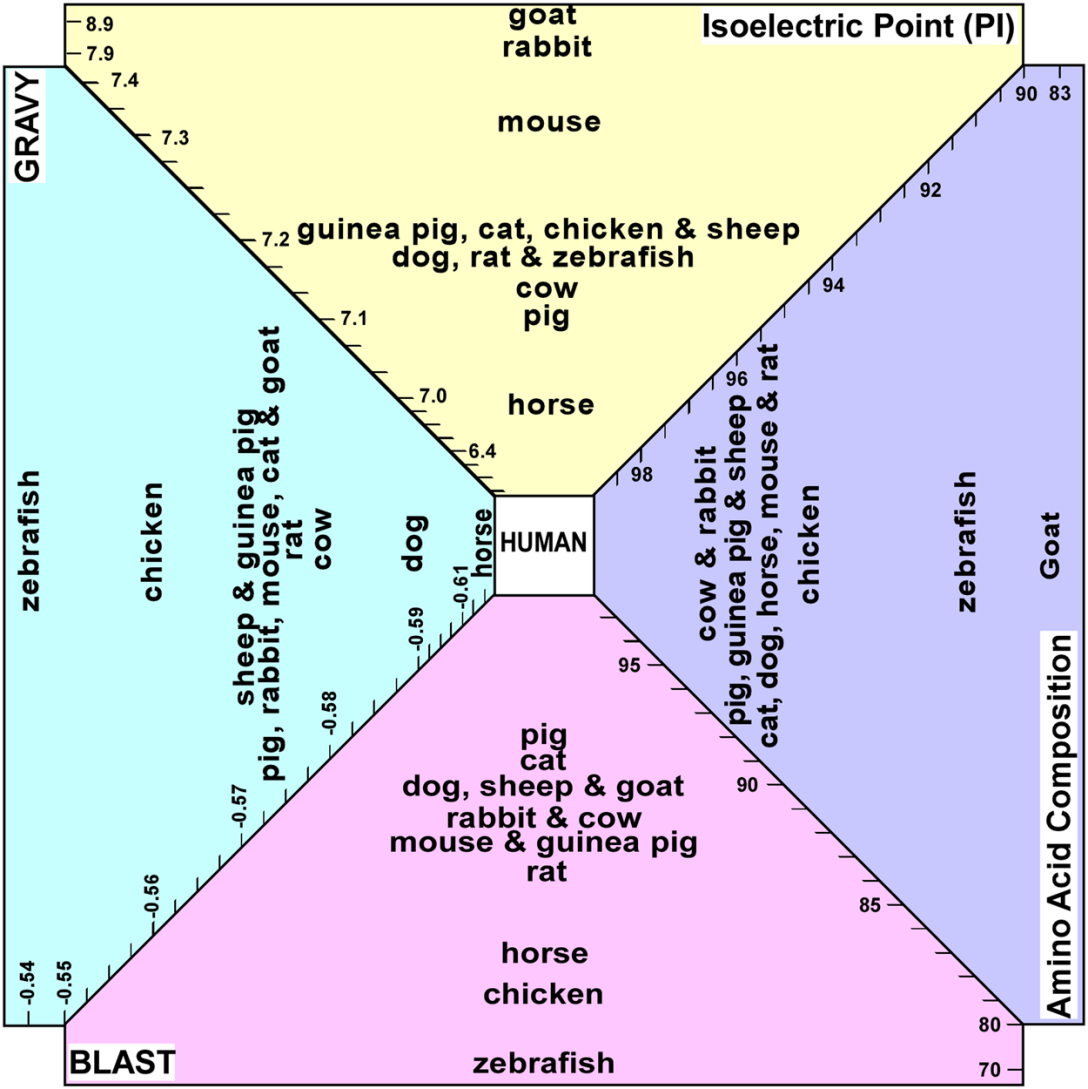
Integrated comparison of similarity for the studied species based on BLAST sequencing, primary amino acid structure, isoelectric point and hydropathicity analysis.

Species that were most commonly used for studies in clinical applications of corneal transplant and also readily available were included: pig (*Sus scrofa*), rabbit (*Oryctolagus cuniculus*), guinea pig (*Cavia porcellus*), chick (*Genus genus*), cat (*Felis catus*), dog (*Canis lupus familiaris*), rat (*Rattus norvegicus*), mouse (*Mus musculus*), bovine (*Bos taurus*), sheep (*Ovies aries*), goat (*Capra hircus*), horse (*Equus callabus*) and zebrafish (*Danio rerio*). Depending on the size and anatomical characteristics, the potential clinical application can vary from full thickness to lamellar grafts, to small plugs for corneal perforations. The corresponding protein for each species was obtained through PUBMED protein database^56^. The most recent sequence of each protein was selected (Supplementary Table 1). If the full sequence was not available, we used the partial sequence, predicted form, or isoforms depending on availability. The amino acid sequence of each protein was compared to human using BLAST algorithm (Basic Local Alignment Search Tool), which gives a percentage of similarity of the compared sequences with respect to those of human. If there were multiple isoforms available, the average percentage of similarity of the isoforms to human was taken. A final weighted score was obtained for each analyzed corneal stromal protein by multiplying the similarity percentage of that specific protein compared to human (obtained from BLAST) by the relative abundance of the protein in the human corneal stroma according to Dyrlund *et al*.^25^. Finally, a total score of similarity to human for each of the analyzed animal species was calculated by averaging all the weighted scores per protein for each of the species (Table 1 and Fig. 1a). In addition, primary amino acid structure (Fig. 2), isoelectric point (PI) (Fig. 3), and grand average of hydropathicity (GRAVY) (Fig. 4), of the proteins with abundance of ≥2% in the corneal extracellular matrix were analyzed (i.e. collagen α-1 (I), α-1 (VI), α-2 (I) and α-3 (VI), decorin, lumican, and keratocan). The amino acid sequence of each protein was obtained from the NIH protein database^56^, using its FASTA tool. These sequences, using the ProtParam tool of ExPASy Bioinformatics Resource Portal, were then analyzed to extract the aforementioned information^57^. The obtained values were compared to those of humans, and final similarity values calculated based on the similarity of each amino acid and the abundance of the protein in the cornea (Fig. 2h). The final GRAVY and PI values were obtained by multiplying the value obtained from the portal by the abundance of each protein in the human corneal stroma (Fig. 3h and 4h). In addition, the similarity comparison of all species based on BLAST sequencing, primary amino acid structure, isoelectric point and hydropathicity analysis was integrated in Fig. 5. We assumed that the relative abundance of each protein in the stroma across all species was similar to humans, as there was no data for the specific animal species analyzed in this work.

## Supplementary information

Supplementary Table 1

## Data Availability

The datasets generated and/or analyzed during the current study are available from the corresponding author upon request.
