# Supplementary material for: Finding an Optimal Corneal Xenograft Using Comparative Analysis of Corneal Matrix Proteins Across Species
Source: Sci Rep. 2019 Feb 12;9:1876. doi: 10.1038/s41598-018-38342-4 (PMC6372616; doi:10.1038/s41598-018-38342-4)
Supplement: Supplementary file 1 — Supplementary Table 1 [file 41598_2018_38342_MOESM1_ESM.pdf]

Finding an Optimal Corneal Xenograft: Comparative Analysis of Corneal Matrix Proteins Across Species.

Sharifi R[1*], Yang Y[1, 2*], Adibnia Y [1, 3], Dohlman CH[1], Chodosh J[1], Gonzalez-Andrades M[1, 4**]

Supplementary Table 1. Taxonomy ID of proteins examined for each species.

	Collagen alpha-1(I) chain (COL1A1)	Collagen alpha-2(I) chain (COL1A2)	Collagen alpha-1(III) chain (COL1A3)	Collagen alpha-1(V) chain (COL1A5)	Collagen alpha-2(V) chain (COL5A2)	Collagen alpha-1(VI) chain (COL6A1)	Collagen alpha-2(VI) chain (COL6A2)	Collagen alpha-3(VI) chain (COL6A3)	Collagen alpha-1(XII) chain (COL12A1)	Decorin	Lumican	Keratocan	Biglycan	MAM domain-containing protein 2 (MCMDC2)	Prolargin	Vimentin
Human	AAH36531.1	P08123.7	AAH28178.1	NP_001839.2	P20908.3	AAH86874.1	AAH65509.1	AAI50626.1	AAI46869.1	AAA52301.	AAA91639.1	AAC16390.1	AAA52287.1	AAH16383.1	NP_958505.1	NP_003371.2
Pig	BAX02568.1	NP_001230584.1	NP_001230226.1	BAE93249.1	NP_001014971.1	NP_001098759.1	XP_013845689.1	BAE93252.1		AAD33862.1 AAD23578.1	NP_001230268.1	XP_013853546.1	Q9GKQ6.2	NP_001231912.1	XP_005656706.1	P02543.2
Rabbit	XP_017204320.1	NP_001182597.1	XP_002712379.1	XP_017194981.1	XP_017195888.1	XP_002712380.1	XP_008265474.2*	XP_008249075.1	Q28902.1	AAB33083.1	NP_001182609.1	XP_002711430.1	NP_001182620.1	Isomers (XP_017197218.1, XP_002708240.1)	XP_002717628.1	XP_002717466.1
Guinea pig	Isomers (XP_013004992.1, XP_013004993.1)	XP_005006094.1 , XP_003475044.2	Isomers (XP_013012877.1, XP_013012878.1, XP_013012876.1 , XP_013012875.1 , XP_013012874.1)	XP_013000445.1 *	XP_013008706.1	XP_003478750.1	Isomers (XP_003463209.2 XP_005000633.1)	Isomers (XP_013012874.1 XP_013012875.1 XP_013012876.1 XP_013012877.1 XP_013012878.1)	XP_012997177.1 XP_003477950.2	ACG50141.1	XP_003475879.1	XP_013014494.1	XP_003462210.1	XP_003472251.1	XP_003474866.1	NP_001166511.1
Chick	P02457.3	NP_001073182.2	NP_990711.2	NP_990438.1	NP_990121.2	XP_015144688.1 XP_004942453.1 XP_015144689.1	NP_990679.1	AAA03201.1	BAA00701.1	CAA45318.1	NP_001263286.1	AAC15506.1		XP_003643063.1	XP_418054.2	NP_001041541.2
Cat	XP_003996748.1, XP_006940277.1	XP_003982813.1	XP_003991010.1	XP_011284013.1	XP_011287018.1	XP_003991011	XP_011284015.1 XP_011284016.1	XP_019694498.1*	Isomers (XP_019686426.1 XP_019686427.1 XP_019686429.1 XP_006931912.1)	XP_003989160.1	XP_003989158.1	XP_006933986.1	XP_019680037.1	Isomers (XP_011286526.1, XP_011286527.1, XP_019671307.1, XP_019671309.1, XP_003995424.2)	XP_006942941.1	XP_003988180.1

Dog	NP_001003090.1	NP_001003187.1	XP_851009.1	NP_001003090.1	XP_013972345.1	XP_005640450.1	XP_013965345.1	NP_001096685.1	XP_013973586.1 XP_005627592.1 XP_539002.2 XP_005627593.1	AAB51245.1	XP_539716.1	XP_005629277.1	AAB51244.1	XP_851100.1, XP_005615934.1	XP_005640817.1	NP_001273952.1
Rat	P02454.5	P02466.3	AAH87039.1	XP_215375.5	NP_604447.1	NP_445940.1	AAH89923.1	Isomers (EDL97135.1, EDL92061.1, EDL92062.1, EDL92064.1, EDL92065.1)	AAB07870.1	AAH83750.1	AAH61878.1	NP_001101557.1	AAA58797.1	EDM13018.1	NP_445837.1	NP_112402.1
Mouse	P11087.4	Q01149.2	AAH28248.1	NP_034063.1	NP_056549.2	NP_031763.2	EDL31842.1	AAC23667.1	NP_001277237.1	P28654.1	EDL21630.1	AAC61257.1	EDL29897.1	NP_777282.1	NP_473418.3	NP_035831.2
Cow	P02453.3	NP_776945.1	P04258.1	NP_001137337.1	AFR46612.1	Isomers (XP_010800138.1 XP_015317726.1)	NP_001068594.1	Isomers (DAA30924.1 , DAA30925.1, DAA30926.1, DAA30927.1)	NP_001193426.1	P21793.2	Q05443.1	O62702.2	AAI18461.1	XP_015320003.1	NP_776859.1	NP_776394.2
Sheep	XP_014954201.1*	XP_004007775.1	XP_004004563.1	XP_004003436.1*	XP_014949583.1*	XP_004004562.1	XP_014947255.1*	AAF79924.1	Isomers (XP_014952772.1 , XP_012037779.1 ,XP_014952773.1)	Q9TTE2.1	XP_012029466.1	XP_012029465.1	NP_001009201.1	: XP_004004372.1 , XP_011978822.1	XP_004013593.1	ABP48145.1
Goat	XP_017900444.1	XP_005678993.1	XP_005675926.1	XP_017907809.1	XP_017911687.1	XP_005675925.2	Isomers (XP_017907820.1 XP_017907828.1)	Isomers (XP_017896193.1 , XP_017896201.1, XP_017896208.1 , XP_017896216.1, XP_013818488.2 , XP_017896230.1)	Isomers (XP_017908356.1 XP_017908357.1 XP_017908359.1)	AEA36044.1	XP_017903107.1	XP_005679867.1	XP_017900688.1	XP_005683777.2	XP_005690429.1	XP_017912644.1
Horse	XP_014592408.1*	NP_001075233.1	AAD13114.1	XP_001488401.2	XP_014591730.1*	XP_001501867.1	XP_014591965.1	XP_014595871.1*	BAB64260.1	AAB92652.1	ABC69292.1	XP_014592227.1 , XP_001492790.2	AAB88305.1	XP_001489371.1	XP_001915309.1	NP_001230074.1
Zebrafish	AAH63249.1	AAH71278.1	NA	XP_009304444.1	ADG36303.1	NP_001139254.1	AAI34052.1	Isomers (XP_017213295.1 , XP_017213296.1, XP_017213297.1, XP_017213298.1)	CAQ03443.1	AAH44206.1	ACU68929.1	AAI15059.1	AAH74062.1	NP_001123869.1	XP_001923590.1	AAC98491.1
